# Supporting Vulnerable Older Adults With Telehealth Through Wellness Calls and Tablet Distribution During COVID-19: Quality Improvement Project

**DOI:** 10.2196/46002

**Published:** 2023-09-11

**Authors:** Sweta Tewary, Nicole Cook, Marie Dezine, Oksana Shnayder, Naushira Pandya

**Affiliations:** 1 Nova Southeastern University Fort Lauderdale, FL United States

**Keywords:** COVID-19, telehealth, older adults, isolation, health education, mental wellness, telemedicine, health literacy, digital health, mental health, social isolation

## Abstract

**Background:**

Loneliness, social isolation, and lack of technical literacy are associated with poorer health outcomes. To help improve social connection during the COVID-19 pandemic, Nova Southeastern University’s South Florida Geriatric Workforce Enhancement Program partnered with a community-based organization to provide educational resources to promote telehealth services.

**Objective:**

This study aimed to provide educational resources to older adults with limited resources and promote the use of telehealth services in this population.

**Methods:**

Through this pilot project, we contacted 66 vulnerable older adults who expressed interest in telehealth support through wellness calls, with 44 participants moving on to participate in tablet usage. All tablets were preloaded with educational information on using the device, COVID-19 resources, and accessing telehealth services for patients, caregivers, and families.

**Results:**

Feedback from wellness assessments suggested a significant need for telehealth support. Participants used the tablets mainly for telehealth (n=6, 15%), to connect with friends and family (n=10, 26%), and to connect with faith communities (n=3, 8%).

**Conclusions:**

The findings from the pilot project suggest that wellness calls and telehealth education are beneficial to support telehealth usage among older adults.

## Introduction

The COVID-19 pandemic has impacted older adults, especially those with chronic conditions. It is estimated that 8 out of 10 deaths related to COVID-19 are reported among older adults aged 65 years and older [1]. In Florida, 83% of total deaths related to COVID-19 are of older adults aged 65 years and older [2]. Many of these deaths have been reported in Miami, Broward, and Palm Beach counties. Of the 1227 people in Florida who tested positive for the virus in early 2020, 38% were aged 60 years or older, and more than half were ≥50 years old [3]. While social distancing measures in community dwellings decreased the risk of spreading COVID-19 among older adults, they triggered several social-emotional barriers among families and providers caring for their loved ones in homes, facilities, and daycare centers. Older adults are less likely to have access to many digital connections younger people use to connect with their loved ones [3]. Loneliness, social isolation, and lack of technical literacy are factors associated with a negative quality of life and poorer health outcomes, especially among homebound older adults.

Older adults are vulnerable to changes ranging from contextual factors to complex social, behavioral, and physical vulnerabilities. The pandemic has profoundly impacted older adults in their ability to receive support including daily routine assistance, medical care, and social and economic well-being. In addition, many older adults are at risk of violence, abuse, neglect, fear of losing loved ones, and caregivers, and isolation impacting mental health. According to Cacioppo and Cacioppo [4], isolated people have a higher risk for depression, cognitive decline, and dementia. Approximately one-quarter of Americans aged 65 years and older are believed to be socially isolated. A report from the National Academies of Sciences and Medicine stated that social isolation was associated with about a 50% increased risk of dementia [5]. Another study suggested socially isolated adults are less likely to use telehealth [6].

Loneliness has further contributed to the fear of forming, maintaining, or sustaining social connections. Loneliness is the feeling of being alone regardless of the amount of social contact [1]. Coyle and Dugan [7] described social isolation as the physical absence of social interactions, including relationship interaction and support from family, friends, or even society [8]. Other authors have described loneliness as a subjective interpretation that may differ based on individual differences. This subjective interpretation is based on the desired and actual social relationship in terms of companionship, connectedness, and intimacy [9,10]. Regardless of the interpretation of the construct, social isolation has been recently added to the list of lifestyle risk factors for untimely mortality rates in line with well-established factors like smoking, obesity, physical inactivity, and air pollution [11]. Recent studies suggest that social connections significantly impact mental and physical health. A 4-year study by Shankar et al [12] that used regression analyses to evaluate data collected from participants with a mean age of 65.6 (SD 9.5) years showed a positive association between isolation and loneliness, as well as a decrease in memory and cognitive function [13].

There has been a massive shift to telemedicine during the COVID-19 pandemic to protect medical personnel and patients. The application of telemedicine has a positive impact on public health emergencies by allowing large numbers of health care providers to offer services when local hospitals and health care centers are unable to meet demand. There has also been growing evidence in patient outcomes when there is an effort to provide telemedicine through an age-friendly approach. Age-Friendly Health System (AFHS) is based on evidence-based principles of the 4Ms: mentation, mobility, medication, and what matters provides care to older adults [14]. A recent review discussed the strengths of telemedicine for medication assessment, frailty review, fall risk, and nutritional requirements. This review also suggests telehealth as an application for chronic disease management.

Telemedicine has changed the way we provide health to everyone including the most vulnerable patients and older adults. Telemedicine has also improved access to health care in rural populations by eliminating the burden of travel arrangements and reducing the cost of medical care [15]. One study suggests that patients' satisfaction in clinical settings improves when providers use telemedicine techniques to meet with patients [16]. Even though telemedicine has demonstrated its applicability in geriatric medicine, its feasibility in supporting homebound older adults through an age-friendly approach is yet to be determined.

It is challenging to provide telemedicine to vulnerable older adults because they have limited digital literacy and access to technology to connect with their medical providers [17]. Many of them are isolated homebound adults with no internet connection. Since older adults are vulnerable and living alone with limited access to resources, social connections, and increased fear, it is important to connect with patients and their loved ones to provide an appropriate education. Ongoing telehealth visits can facilitate interactions with providers and patients with reduced exposure to the virus while still being connected with health care services. To help improve social connections during the pandemic, Nova Southeastern University’s South Florida Geriatric Workforce Enhancement Program collaborated with primary care clinics and a local community–based organization to provide educational support to older adults living in Broward County. The purpose was to (1) assess wellness needs and barriers to access to care, (2) provide educational resources, and (3) promote telehealth services.

## Methods

### Overview

Our project population included a total of 196 adults aged 65 years and older who were eligible for telehealth visits. Inclusion for eligibility was assessed through the designation of underprivileged or vulnerable populations as per the Broward County Distressed Community Index. Individuals from the community-based organization were referred through the case manager working with the homebound populations. All participants were contacted 3 times by the project team; however, our final sample included 66 homebound participants who agreed to participate in the 12-month project period from both a university clinic (UC) as well as a community-based organization (CBO).

The project was conducted in 2 phases. The first phase involved conducting wellness calls for existing patients of a UC and homebound medically underserved individuals receiving services through a CBO. The timeframe for wellness calls for both organizations was July to December 2021. Questions in the wellness calls survey were designed and adapted by the project team from a questionnaire assessing the impact of COVID-19 on older adults [18]. Specifically, the questions addressed the diagnosis of COVID-19, access to masks, access to telehealth devices, and so forth. Access to the internet and experience with other technology devices were also explored. The wellness calls with 32 UC participants were conducted by the technical support coordinator and GWEP-affiliated health professional students who received Research Electronic Data Capture (REDCap; Vanderbilt University) training through multiple Zoom (Zoom Technologies Inc) sessions on sensitivity, patience, consistency, and best practices for conducting surveys with older adults via phone. Monthly schedules were established by the project team to follow up on survey questions and training modules for telehealth support.

While completing the wellness survey with the participants, the project team was informed about several questions being repeated after the initial administration to the participants. In order to reduce duplication and improve participation without fatigue, we identified fields or questions and edited with the project team to improve face validity while reducing potential duplication in the wellness call survey. Due to the limited timeframe, the survey was modified and rolled out based on early and rapid, verbal feedback from participants. The final survey was edited and updated in October 2020 to remove the duplication from the initial version and administered to CBO participants. Data collection was conducted in REDCap, a mature, secure web app for building and managing web-based surveys and databases (see Multimedia Appendix 1).

The second phase of the project involved providing telehealth education to participants interested in receiving a tablet for a period of 6 months to help with access to telehealth services during the pandemic. All participants who expressed interest in telehealth support through the wellness survey were assessed for interest in receiving a tablet. All participants were provided with internet-enabled tablets mailed to their home addresses for 6 months from January to July 2021. Tablets were preloaded with educational materials in written and video format. Information was focused on step-by-step tablet usage; nearby COVID-19 resources; and accessing telehealth services for patients, caregivers, and their families. At the end of the 6-month period, the assessment of tablet usage was conducted over the phone via a short feedback questionnaire (see Multimedia Appendix 2).

### Ethical Considerations

Ethical review and approval were waived for this study due to the nature of the study. The study was a quality improvement project and nonhuman subject research (IRB#2020-440). The protocol does not require review or approval by an institutional review board (IRB) because its procedures do not fall within the IRB’s jurisdiction based on 45 CFR 46.102.

### Data Analysis

Data were analyzed using descriptive statistics and summaries of biweekly phone conversations with project participants.

## Results

### Phase 1: Wellness Calls Survey Results

#### Descriptive Statistics

All 196 participants were contacted; however, only 66 participants chose to receive telehealth support and complete the wellness survey. Others were either not available, not interested, or wanted to be contacted later. Patients who responded to the wellness survey had a mean age of 74 (SD 9.09) years. Thirty (45%) respondents shared that they live alone and 37 respondents (56%) informed that they had a family member or friend providing them with social support. Most of the respondents were non-Hispanic (see Table 1). During the discussion based on the pandemic, 56 (85%) participants reported they are currently being seen by their primary care providers. Regarding barriers, 17 (26%) participants expressed difficulty in access to medical care. When asked if there are any concerns about using telehealth services for doctor appointments, 39 (59%) participants stated they do not have any concerns. In general, 26 (40%) participants stated that before COVID-19 they often felt isolated. Specifically, 43 (65%) participants reported feeling isolated from friends and families during the pandemic. When screened for telehealth services and support, 56 (85%) participants did not reject scheduling a telehealth visit with the physician. Thirty-six participants (55%) could use live video without the assistance of others (see Table 2).

**Table 1:** Demographic data of patients who participated in the wellness survey (N=66).

Demographics			Values
**Gender,** **n (%)**			
	Male	20 (30)	
	Female	45 (68)	
	Prefer not to respond or other	1 (1)	
Age (years), mean (SD)			74 (9.09)
**Race,** **n (%)**			
	Non-Hispanic	36 (55)	
	Black Caribbean	7 (11)	
	Hispanic	2 (3)	
	African American	16 (24)	
	Prefer not to respond or other	5 (8)	

**Table 2:** Wellness survey responses (N=66).

Category and survey questions		Responses, n (%)		
		Yes or often	No or never	Sometimes
**Level of isolation**				
	Do you live alone?	30 (45)	36 (54)	N/A^a^
	Before the stay-at-home order, how often did you feel isolated from others?	7 (11)	38 (57)	19 (29)
	Currently, how often do you feel isolated from others?	18 (27)	22 (33)	25 (38)
	How often do you feel that you lack companionship?	11 (17)	26 (39)	29 (44)
**COVID-19: safety and wellness**				
	Have you been diagnosed with COVID-19?	4 (6)	61 (92)	1 (1)
	Does the patient have a regular care provider?	56 (85)	7 (11)	2 (3)
	How much difficulty do you have with getting routine medical care because of the COVID-19 pandemic?	12 (18)	49 (74)	5 (7)
	How much difficulty do you have obtaining the medications that you need because of the COVID-19 pandemic?	1 (1)	59 (89)	5 (7)
**Education and resources**				
	Have you had difficulty obtaining a face covering or mask?	38 (57)	27 (41)	1 (1)
	Do you currently wear a face covering or mask when near other people	66 (100)	0 (0)	0 (0)
	Do you follow the social distancing and hygiene guidelines for COVID-19? (registered nurse goes over protocols)	65 (98)	1 (1)	0 (0)
**Telehealth and support**				
	Prior to the COVID-19 pandemic, did you use live video on electronic devices (such as a computer or smartphone) to communicate with friends and family?	35 (53)	29 (44)	0 (0)
	Have you rejected scheduling a telehealth visit offered by your physician during the COVID-19 pandemic?	10 (15)	56 (85)	0 (0)
	Have you had a telehealth visit with your physician (instead of in person) due to the COVID-19 pandemic?	29 (44)	36 (54)	0 (0)
	Can you use live video without the assistance of others?	36 (54)	27 (41)	2 (3)

^a^N/A: not applicable.

### Phase 2: Telehealth Support—Tablet Usage

#### Demographics

Patients who received tablets included 44 individuals from CBO as well as UC, with a mean age of 72 (SD 9.07) years. Most of the participants were female (see Table 3). In addition, 7 (16%) participants identified themselves with some form of disability, and 6 (14%) participants expressed being part of the lesbian, gay, bisexual, transgender, queer or questioning, intersex, asexual, and more (LGBTQ+) community.

**Table 3:** Demographic data of patients who received tablets (n=44).

Demographics			Values
**Gender, n (%)**			
	Male	15 (34)	
	Female	29 (66)	
Age (years), mean (SD)			72 (9.07)
**Race, n (%)**			
	Hispanic	5 (11)	
	African American	23 (52)	
	White	12 (27)	
	Prefer not to respond or other	4 (9)	

#### Responses on Tablet Usage

This section describes responses to open-ended questions asked as part of telehealth support after 6 months of tablet use. Participants were contacted 3 times to ensure feedback on tablet usage. Questions were structured around tablet usage and the 4Ms of AFHS (mentation, mobility, medications, and what matters).

Among the 44 patients who received the tablet, 39 (89%) participants used the tablet in some capacity, and 10 (23%) participants reported using it daily. In total, 6 (15%) participants used it for telehealth; 7 (18%) for research; 8 (20%) for entertainment; 10 (26%) to connect with friends and family; 3 (8%) to connect with faith/ministries; and 4 (57%) with other uses including shopping, news, and apps (see Table 4). Additionally, 16 (36%) participants found the education materials impactful. Nearly all participants reported comfort with the tablet’s availability for socialization and searching the internet for health-related information.

**Table 4:** Use of tablets among participants (n=39).

Category	Respondents^a^, n (%)
Internet (general)	10 (26)
Social connection (eg, Zoom and video calls)	10 (26)
Entertainment (eg, movies, books, games)	8 (20)
Applications (eg, Facebook)	7 (18)
Researched medical information	7 (18)
Telehealth	6 (15)
Shopping, news, and apps	4 (10)
Faith-based services	3 (8)
No response or reported no usage	12 (31)

^a^Categories are duplicative of participants.

#### Responses on AFHS and the 4Ms

##### Overview

Approximately, 32 (73%) participants responded to feedback questions about tablet usage. Even though participants used tablets for multiple needs, the use of technology was central and exciting for many older adults, especially during the pandemic. The following sections describe the themes from an AFHS perspective and summarize how participants found comfort in using the tablet for what matters to them most.

##### Mentation

The participants expressed that the tablets helped them with socialization with family, friends, and extended networks. One participant who was visually blind used the tablet for audiobooks and to connect with her family. Another participant, who identified himself as LGBTQ+, discussed his learning disability and expressed an improvement in isolation and health issues after receiving the tablet. A female patient with dementia who is bedbound indicated limited knowledge of tablet usage but with training found the tablet very helpful. Her family members assisted her with tablet usage, translation of educational resources, telehealth visits, and making social connections.

##### Medication Management

The participants reported that the tablets helped them with telehealth appointments and researching medical information. One wheelchair-bound participant from the LGBTQ+ community indicated that he had transportation issues and the project helped him learn to set up telehealth appointments and order medications through the tablet. He enjoyed the conversations about healthy diet and exercise and used the tablet to research medical information, contact stores, refill medication at his local pharmacy, and schedule a telehealth appointment.

##### Mobility

We also explored if the tablet helped the participants in improving their mobility. One participant expressed how their physical therapist included navigation on the tablet as a means of improving dexterity. Another patient shared how the tablet was useful to assist her with her occupational therapy.

##### What Matters

When asked about what matters most, participants expressed a need for social support. The tablet was used to connect with family, friends, church, and loved ones. It was also useful for entertainment, shopping, and accessing education sessions through Zoom provided through the university as well as their local Church.

## Discussion

### Principal Findings

The results of the wellness survey identified a need for social connectedness and telehealth support. Many patients did not respond to telehealth calls due to survey fatigue, marketing spam, and lack of interest. Participants who responded to the telehealth calls found the education useful and were interested in learning about telehealth services. Through the tablet distribution, the project was successful in delivering digital education on telehealth services, COVID-19 resources, and how to access a telehealth provider. The participants were excited to receive the tablets with an internet connection and indicated that it was helpful with social isolation and social connectedness as identified in previous studies [19,20]. They also appreciated the wellness calls conducted during the project as they helped them reflect on their health needs, facilitate open dialogue, and assess any concerns. Biweekly phone conversations helped provide telehealth support, especially during the pandemic. Through the pilot project, we learned that there was a need for educational resources for telehealth services, access to an internet connection, telehealth device, and phone connection. The results of the project are consistent with the gaps identified in the literature review about the lack of telehealth education, access, and loneliness contributing to some of the barriers associated with providing telehealth [17]. Overall, the results indicate the importance of the telehealth initiative.

### Limitations

This pilot project had a few limitations. Many respondents were not interested in participating in wellness calls. Hearing issues, lack of device, internet, and phone connection were some of the reasons cited by the respondents. Respondents who participated expressed their concern about limited COVID-19 testing, lack of telehealth education, transportation, lack of face masks, and issues with medication refills. Participants who were provided with tablets also reported some concerns. For example, older adults encountered challenges in setting up telehealth appointments discussed during biweekly calls. Some of these are (1) each medical provider has a certain way of setting up their telehealth appointments either through an application or link, and this was difficult for some seniors to understand and access; (2) individual pharmacies also have their own apps for electronic medication prescribing, and this was difficult for some seniors to understand resulting in multiple phone calls to pharmacies, and (3) some cited concerns about the financial impact of maintaining an internet connection.

### Conclusions

The pilot project demonstrated that many homebound older adults can be educated to use technology (tablets) to connect with health care providers, friends, family, and other support systems if appropriate guidance is provided to support technology adoption. Despite its short duration, this program helped participants with some of their health and wellness needs via telehealth applications and allowed access to meaningful social interactions with friends and family during the pandemic. The project also demonstrated that distributing health education information via tablets can be an effective strategy for facilitating telehealth visits. However, future studies should test the usability of tablets for a longer duration. With limited digital literacy and newer technologies, studies should continue the evaluation of technology adoption interventions for homebound seniors in postpandemic settings.

## Appendix

Multimedia Appendix 1 Wellness survey.

Multimedia Appendix 2 Tablets usability feedback questionnaire.

## Abbreviations

AFHS: Age-Friendly Health System
CBO: community-based organization
IRB: institutional review board
LGBTQ+: lesbian, gay, bisexual, transgender, queer or questioning, intersex, asexual, and more
REDCap: Research Electronic Data Capture
UC: university clinic

## Data Availability

Due to confidentiality restrictions, the data that support the findings of this project are not available for public access.
