# Supplementary material for: Supporting Vulnerable Older Adults With Telehealth Through Wellness Calls and Tablet Distribution During COVID-19: Quality Improvement Project
Source: JMIR Form Res. 2023 Sep 11;7:e46002. doi: 10.2196/46002 (PMC11042788; doi:10.2196/46002)
Supplement: Multimedia Appendix 1 [file formative_v7i1e46002_app1.pdf]

Wellness Calls 2020

On approximately March 15th the State of Florida issued the 'safer-at-home'/'stay-at-home' order to slow the spread of Covid-19. This extended 'social distancing' guidelines set in place a few days prior. The purpose of this survey is to understand your experiences with technology, behaviors and physical/mental health before, and now during, the 'safer- at-home' order.

The survey is completely anonymous. Results will be used to help improve Zoom delivery to lifelong learners and to help inform on what strategies and characteristics may be beneficial to other older adults during social distancing and stay at home orders.

Date (M/D/Y) ______ M-D-Y

Gender
- ○ Male
- ○ Female
- ○ Neither male or female/not identified

Name of person conducting wellness calls ______

Patient belongs to which clinic/organization?
- ☐
- ☐
- ☐
- ☐
- ☐

Age of client ______

Client race/culture?
- ○ white non Hispanic
- ○ Black Caribbean
- ○ Black African American
- ○ Hispanic
- ○ Prefer not to response/other

Do you live alone?
- ○ Yes
- ○ No

**If you do not live alone, who do you live with? (spouse, child, other relative, friend, professional attendant)**	
**Who is providing you with social support during the outbreak? (check all that apply)**	☐ someone I live with<br>☐ friend or family who comes by my place<br>☐ friend or family who I talk with on the phone [or video chat]<br>☐ other<br>☐ professional attendant<br>☐ caregiver/volunteer<br>☐ I do not have support
**Before the stay at home order, how often did you feel isolated from others?**	◯ Never<br>◯ Sometimes<br>◯ Often
**Currently, how often do you feel isolated from others?**	◯ Never<br>◯ Sometimes<br>◯ Often
**Are you going out in public during the current Covid-19 pandemic? (check all that apply)**	☐ Homes of others<br>☐ Medical Provider Visit<br>☐ Grocery store<br>☐ Pharmacy<br>☐ Restaurant<br>☐ Leisure activity<br>☐ Work<br>☐ Volunteer<br>☐ Other<br>☐ Not going out at all
**Do you follow the social distancing and hygiene guidelines for Covid-19? (RN go over protocols)**	◯ Yes<br>◯ No

Do you currently wear a face covering or mask when near other people	◯ Yes<br>◯ No<br>◯ Sometimes
Prior to the COVID-19 pandemic, did you use live video on electronic devices (such as a computer or smart phone) to communicate with friends and family?	◯ Yes<br>◯ No
Do you currently use live video (Facetime/Skype/Zoom) on electronic devices (such as a computer or smart phone) to stay connected with friends and family because of Covid-19?	◯ Yes<br>◯ No<br>◯ Sometimes
Can you use live video without the assistance of other?	◯ Yes<br>◯ No<br>◯ Sometimes
Do you have any concerns about using telehealth services for your doctor appointments?	
Do you have difficulty with getting routine medical care because of the Covid-19 pandemic?	◯ Yes<br>◯ No<br>◯ Sometimes
Have you rejected scheduling a telehealth visit offered by your physician during the Covid-19 pandemic	◯ Yes<br>◯ No
Have you had a telehealth visit with your physician (instead of in person) due to COVID-19 pandemic?	◯ Yes<br>◯ No
Do you have difficulty with obtaining the medications that you need because of the Covid-19 pandemic?	◯ Never<br>◯ Sometimes<br>◯ Often
Do you have difficulty with obtaining the food that you need because of the Covid-19 pandemic?	◯ Never<br>◯ Sometimes

	◯ Often
**Have you been diagnosed with COVID-19?**	◯ Yes<br>◯ No<br>◯ Not Sure
**Were you hospitalized for COVID-19?**	◯ Yes<br>◯ No
**How often do you feel that you lack companionship?**	◯ Never<br>◯ Sometimes<br>◯ Often
**During the 'stay at home' order, what strategy/activity/hobby has been most helpful to you in terms of staying physically healthy?**	
**Does the patient have a regular care provider?**	◯ Yes<br>◯ No<br>◯ Not sure
**Any other notes please write here:**	

Submit
