# Supplementary material for: Supporting Vulnerable Older Adults With Telehealth Through Wellness Calls and Tablet Distribution During COVID-19: Quality Improvement Project
Source: JMIR Form Res. 2023 Sep 11;7:e46002. doi: 10.2196/46002 (PMC11042788; doi:10.2196/46002)
Supplement: Multimedia Appendix 2 [file formative_v7i1e46002_app2.pdf]

Telehealth Care Among Older Adults – Tablets Usability Feedback Questionnaire

Have you been able to turn the tablet on and use it?

- ☐ Yes
- ☐ No
- ☐ Not Sure

How have you used the tablet?

- ☐ Shopping
- ☐ Internet/Research (Medical)
- ☐ Entertainment/Games
- ☐ Connect with Family & Friends
- ☐ Telehealth
- ☐ Ministries
- ☐ News
- ☐ Apps
- ☐ No response
- ☐ Other
- ☐ Not At All

On average, how many times in the past week have you used the tablet?

- ☐ Daily/Regularly
- ☐ Occasionally
- ☐ Rarely
- ☐ Not At All
- ☐ No response

Although our program is ending in July, would you prefer to keep the tablets?

- ☐ Yes
- ☐ No
- ☐ No response/ Did not specify

Was the education impactful?

- ☐ Yes
- ☐ No
- ☐ No response/ Did not specify
- ☐ Other

Impact on Socialization?

- ☐ Yes
- ☐ No
- ☐ No response/ Did not specify
- ☐ Other

Provide feedback on the following:
(open-ended questions)

- ☐ Mentation
- ☐ Mobility
- ☐ What Matters
- ☐ Medication
